# Effects of *Anma* therapy (Japanese massage) on health-related quality of life in gynecologic cancer survivors: A randomized controlled trial

**DOI:** 10.1371/journal.pone.0196638

**Published:** 2018-05-03

**Authors:** Nozomi Donoyama, Toyomi Satoh, Tetsutaro Hamano, Norio Ohkoshi, Mamiko Onuki

**Affiliations:** 1 Department of Health, Faculty of Health Sciences, Tsukuba University of Technology, Tsukuba, Ibaraki, Japan; 2 Department of Obstetrics and Gynecology, University of Tsukuba, Tsukuba, Ibaraki, Japan; 3 P4 Statistics Co. Ltd., Setagaya-ku, Tokyo, Japan; Public Library of Science, UNITED KINGDOM

## Abstract

**Objectives:**

*Anma* therapy (Japanese massage therapy, AMT) significantly reduces the severity of physical complaints in survivors of gynecologic cancer. However, whether this reduction of severity is accompanied by improvement in health-related quality of life is unknown.

**Methods:**

Forty survivors of gynecologic cancer were randomly allocated to either an AMT group that received one 40-min AMT session per week for 8 weeks or a no-AMT group. We prospectively measured quality of life by using the Japanese version of the European Organization for Research and Treatment of Cancer QLQ-C30 version 3.0 (EORTC QLQ-C30) at baseline and at 8-week follow-up. The QLQ-C30 response rate was 100%. Hospital Anxiety Depression Scale (HADS), Profile of Mood States (POMS), and Measure of Adjustment to Cancer were also prespecified and prospectively evaluated.

**Results:**

The QLQ-C30 Global Health Status and Quality of Life showed significant improvement at 8 weeks (*P* = 0.042) in the AMT group compared with the no-AMT group, and the estimated mean difference reached a minimal clinically important difference of 10 points (10.4 points, 95% CI = 1.2 to 19.6). Scores on fatigue and insomnia showed significant improvement in the AMT group compared with the no-AMT group (*P* = 0.047 and 0.003, respectively). There were no significant between-group improvements in HADS anxiety and depression scales; however, POMS-assessed anger-hostility showed significant improvement in the AMT group compared with the no-AMT group (*p* = 0.028).

**Conclusions:**

AMT improved health-related quality of life in gynecologic cancer survivors. AMT can be of potential benefit for applications in oncology.

## Introduction

Cancer survivors experience adverse effects years into survivorship [1]. In many studies, the most prevalent physical symptoms reported in cancer survivors are fatigue [1–4], pain [2–4], stress [1,2,4], insomnia [1,2,4], weight gain [1], and lymphedema [4], and the most prevalent psychological symptoms reported are depression [2–6] and anxiety [3–6]. Survivors struggle with symptom burden long into survivorship [5] and cancer survivors experience fear of cancer recurrence [1,7,8]. Thus, the physical symptoms and psychological state of cancer survivors during survivorship might significantly affect quality of life (QOL).

*Anma* massage therapy (Japanese massage therapy, AMT) is a popular form of complementary and alternative medicine in Japan. Based on anecdotal information, it has long been used to relieve physical and psychological complaints in healthy persons as well as in persons with cancer-related symptoms. However, until recently, scientific evidence of the effectiveness of AMT for patients with cancer or cancer survivors has been lacking.

To examine the physical and psychological effects of AMT in survivors of gynecologic cancer, after a preliminary study [9], we designed [10] and conducted [11] a randomized, two-arm, parallel-group, controlled trial over a two-year period. In this study, we confirmed that AMT significantly reduces severity of physical complaints in survivors of gynecologic cancer. AMT also changes the values of certain biochemical markers, possibly by inhibiting the sympathetic nervous system [11]. However, whether this reduction in severity is accompanied by improved health-related QOL (HR-QOL) is not known. Therefore, here we report the effect of AMT on HR-QOL outcomes for survivors of gynecologic cancer.

## Materials and methods

### Trial registration

On October 12, 2012, this trial was registered with the UMIN Clinical Trials Registry as application UMIN000009097: Effects of continuous traditional Japanese massage therapy (*Anma* therapy) for cancer survivors: a randomized controlled trial (https://upload.umin.ac.jp/cgi-open-bin/ctr/ctr.cgi?function=brows&action=brows&type=summary&recptno=R000010670&language=E). The CONSORT checklist for this trial is available in S1 Checklist.

### Ethics statements

The Medical Ethics Committee of Tsukuba University of Technology, Japan, approved this trial on September 27, 2012 (Approval No. 5). Participants were recruited by trial gynecologists working at another hospital and were provided with trial information (oral and written) at the coordinating office of Tsukuba University of Technology. They subsequently submitted a written consent form to participate in the trial by hand or via facsimile. Submission of a consent form was considered to be enrolment. This trial was conducted according to the principles of the Helsinki Declaration.

### Study overview

Recruitment began on October 13, 2012. The first participant’s trial began on November 2, 2012 and the last participant’s final 8-week follow-up session was on November 1, 2014. As described previously [10,11], we enrolled in this trial 40 participants who met the following inclusion criteria: (a) a history of uterine-cervical, endometrial, ovarian, fallopian-tubal, or peritoneal cancer; (b) no recurrence of such cancer for ≥ 3 years; (c) age ≥ 20 years; and (d) confirmed eligible for the trial by the gynecologist responsible for the patient.

After enrollment was completed, randomization was done with an allocation sequence generated by block randomization by the trial statistician. However, allocation adjustment factors were not set in the trial due to insufficient information about factors influencing the effectiveness of AMT. Before commencement of the trial, a table of randomized assignment was created by the same statistician and managed by two employees at the trial’s coordinating office; participants were randomly allocated to an AMT group or no-AMT group.

AMT treatment involved a once-weekly 40-min AMT session for 8 consecutive weeks. According to AMT protocol [11], AMT participants received a full-body massage that utilized standard AMT techniques [12] and focused on specific locations related to their physical complaints. All outcomes were assessed before the first session (pre-session, baseline) and before the final session (8-week follow-up) to verify the effects of 8 consecutive weeks of weekly AMT. Some outcomes were also assessed after the first session (post-session) to confirm the immediate effects of single-session AMT. No-AMT participants did not receive AMT and were followed as usual by their medical doctors, although on the first day, they met with a massage therapist for a 40-min semi-structured chat intervention. The chat intervention included self-disclosure [13], positive thinking [14], and use of a positive feedback method. This chat protocol was previously reported in detail [11]. All outcomes were assessed before the chat session (pre-session, baseline) and some outcomes were also assessed again after the session (post-session). On the final day of the 8-week trial, the no-AMT participants returned to the office for assessment (8-week follow-up) and a single 40-min AMT session as a gift for participating in the study.

### Assessments

We prespecified four instruments to clarify the effects of AMT on patient-reported outcomes: the Japanese versions of the European Organization for Research and Treatment of Cancer QLQ-C30 version 3.0 (EORTC QLQ-C30), Hospital Anxiety Depression Scale (HADS), Profile of Mood States (POMS), and Measure of Adjustment to Cancer (MAC).

We assessed HR-QOL in patients using the Japanese version of the EORTC QLQ-C30 version 3.0. The reliability and validity of this scale has been established and it has been used internationally in a wide range of cancer clinical trials [15]. This scale is a 30-item self-reported Likert method questionnaire that is divided into three subcategories: global health status/QOL (2 items); functional scales, including five subscales (physical functioning, 5 items; role functioning, 2 items; emotional functioning, 4 items; cognitive functioning, 2 items; and social functioning, 2 items); and symptom scales, including nine subscales (fatigue, 3 items; nausea and vomiting, 2 items; pain, 2 items; dyspnea, 1 item; insomnia, 1 item; appetite loss, 1 item; constipation, 1 item; diarrhea, 1 item; and financial difficulty, 1 item). Linear transformation was applied to each subscale to standardize the raw score so that scores range from 0 to 100, with higher scores representing better levels of global health status and functioning and lower scores representing a worsening of symptoms [16]. In this study, we regarded a difference of 10 points or more on the QLQ-C30 score as clinically meaningful [17,18].

The 14-item HADS scale was developed to measure anxiety (7 items) and depression (7 items) in patients with cancer, utilizing a 4-point Likert scale ranging from 0 to 3, with lower scores representing lesser degrees of anxiety/depression [19]. The scale’s reliability and validity has been established for use in Japanese patients [20].

The brief 30-item Japanese version of the original 65-item POMS scale developed by McNair et al. [21] assesses temporal mood states that change according to condition and allows simultaneous assessment of six subscales: tension-anxiety, depression-dejection, anger-hostility, vigor, fatigue, and confusion. Questions were decreased to 30 items to reduce subject burden and allow assessment of changes in mood and emotion over a long time window. The brief version’s reliability and validity has been established and found to be capable of obtaining the same results as the 65-item original version [22]; therefore, it is used widely in many study fields in Japan. In the brief version, each subscale is evaluated with five items on a 5-point Likert scale (0 to 4). Moreover, a total mood disturbance score can be calculated by subtracting the vigor score from the sum of the remaining five subscales. The higher the vigor score and lower the other five subscale scores and total mood disturbance score, the better the mood state.

The MAC scale is a 40-item questionnaire developed by Watson et al. [23] to measure coping responses in patients with cancer. The reliability and validity of the Japanese version has been established [24]. This scale utilizes a 4-point Likert scale (1 to 4) and comprises five subscales on coping styles: fighting spirit (16 items), helplessness/hopelessness (6 items), anxious preoccupation (9 items), fatalism (8 items), and avoidance (1 item). The higher the score in fighting spirit, the more patients tend to exhibit that coping style; whereas the lower the score of the other subscales, the more patients tend to exhibit the relevant coping style.

### Statistical methods

Statistical analysis including how the sample size was determined has been described previously [10,11]. In this study, we defined a modified intention-to-treat (mITT) analysis set, which consisted of all participants who were treated at least once in the AMT arm and all participants who attended the single chat intervention in the no-AMT arm.

For the summary of baseline characteristics, categorical data are presented as frequencies and percentages. Continuous data are presented as medians and ranges. Continuous variables were compared using the Mann-Whitney U test. Categorical variables were examined using Pearson's chi-square test with continuity correction.

All scales were summarized using medians and interquartile ranges. We compared AMT and no-AMT group scores using the Mann-Whitney U test for changes in scores from pre-session measurements to 8-week follow-up measurements. Exact *p*-values were calculated to address issues with small sample sizes and tied scores. We also calculated two-sided 95% exact Hodges-Lehmann confidence intervals for median differences between groups. The mean difference of Global Health Status/QOL in EORTC QLQ-C30 scale and its 95% confidence interval was calculated and compared to a minimal clinically important difference of 10 points [17,18]. We visually confirmed the normality of the Global Health Status/QOL changes by using Q-Q plots. We also applied the same analyses to the POMS scales to examine changes in scores from pre-session to post-session.

All *P*-values were two-sided, and the level of statistical significance was set at 0.05. Multiplicity issues were not considered. SAS 9.4 (SAS Institute, Cary, NC) was used for all analyses.

## Results

### Baseline characteristics

**Fig 1:**
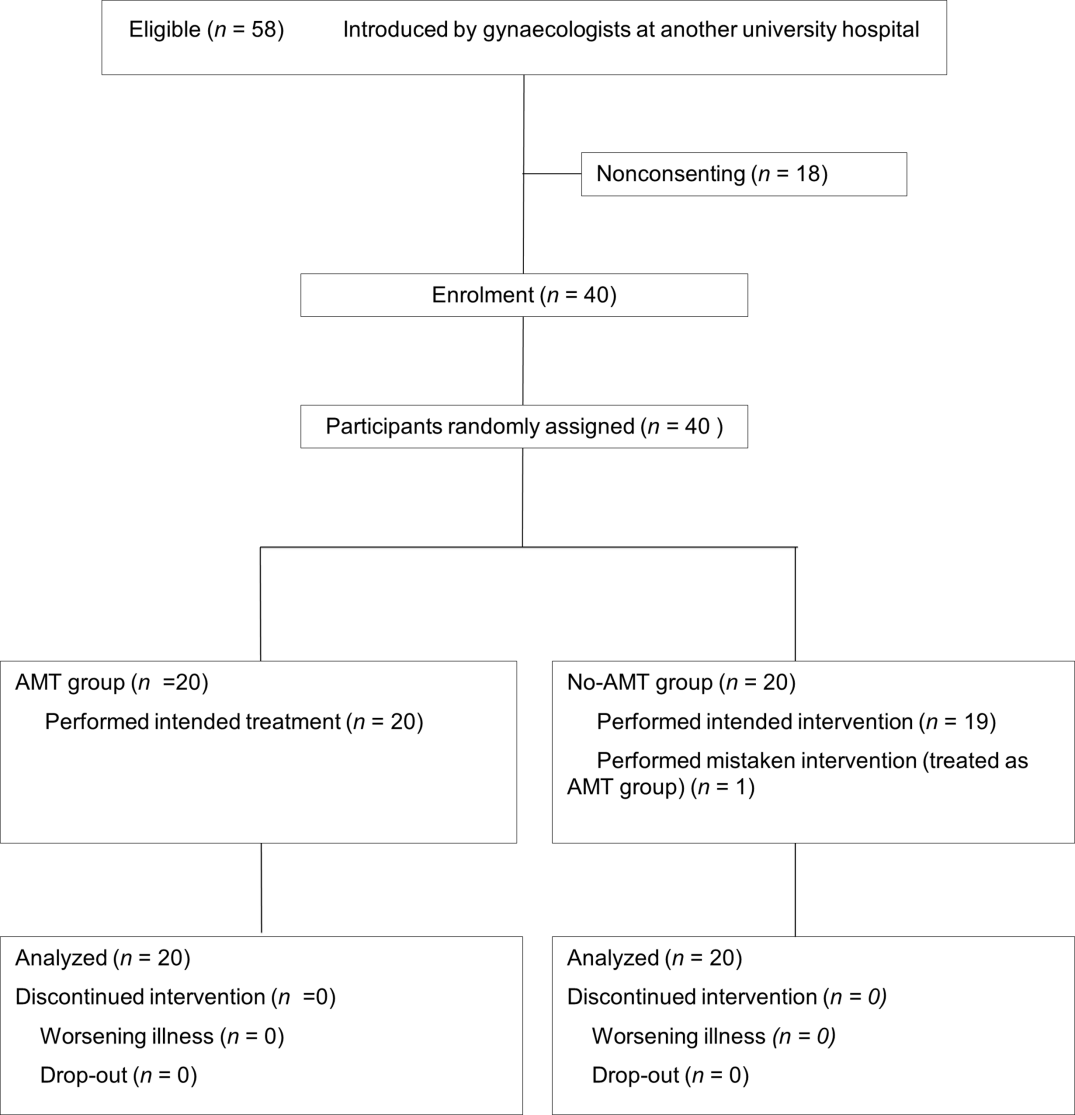
CONSORT flowchart of participant recruitment [11]. AMT: *Anma* massage therapy.

Among 58 eligible participants, we enrolled 40 who submitted the consent form. Twenty participants were randomized to the AMT group and 20 to the no-AMT group. One participant who had been randomized to the no-AMT group received AMT intervention (Fig 1 [11], S1 Fig). Demographic and clinical variables were well-balanced between the AMT and no-AMT groups [11] (Table 1). All participants completed their interventions and provided responses.

**Table 1:** Demographics and baseline characteristics of cancer survivors.

Characteristic	AMT (*n* = 20)	No AMT (*n* = 20)	*P-*value^a^
Age, median (range)	53.0 (40–69)	55.5 (41–75)	0.600
Age at cancer onset, years, median (range)	42.5 (33–59)	43.5 (26–70)	0.677
Duration from cancer onset, years, median (range)	8.3 (3.1–21.3)	8.6 (3.2–27.8)	0.798
Site of gynecologic cancer			
Uterine cervix	11 (55%)	14 (70%)	0.340
Endometrium	7 (35%)	3 (15%)	
Ovary	2 (10%)	3 (15%)	
FIGO stage			
I	13 (65%)	15 (75%)	0.730
II-IV	7 (35%)	5 (25%)	
Surgery			
Yes	20 (100%)	17 (85%)	0.230
No	0 (0%)	3 (15%)	
Lymph node dissection			
Yes	16 (80%)	14 (70%)	0.715
No	4 (20%)	6 (30%)	
Chemotherapy			
Yes	6 (30%)	5 (25%)	>0.999
No	14 (70%)	15 (75%)	
Radiotherapy			
Yes	8 (40%)	5 (25%)	0.500
No	12 (60%)	15 (75%)	

AMT = *Anma* therapy.

^a^*P*-values were calculated by Mann-Whitney U test for continuous variables, Pearson’s chi-square test for categorical variables, and continuity corrections were performed for two-by-two tables.

#### EORTC QLQ-C30

**Fig 2:**
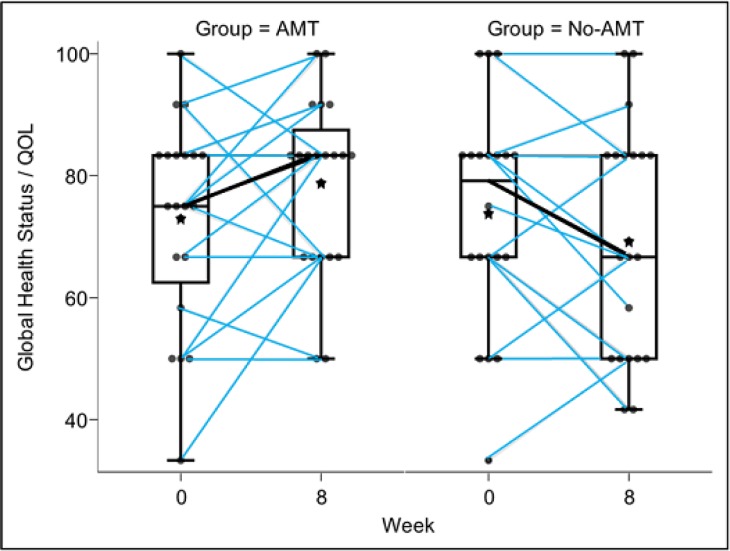
Changes in Global Health scores measured by using the EORTC QLQ-C30. AMT = *Anma* massage therapy.

On the Global Health Status/QOL scale, the AMT group compared with the no-AMT group showed significant improvement at 8 weeks (median scores, from 75 to 83 and from 79 to 67, respectively; *P* = 0.042), and the estimated mean difference reached a minimal clinically important difference of 10 points (10.4 points, 95% CI = 1.2 to 19.6) (Fig 2, Table 2). In functioning scales, we found no significant between-group differences (Table 2). In symptom scales, scores on fatigue and insomnia showed significant improvement in the AMT group compared with the no-AMT group at 8 weeks (median scores of fatigue, from 33 to 28 and from 22 to 33, respectively [*P* = 0.047]; median scores of insomnia, from 33 to 0 and from 0 to 17, respectively [*P* = 0.003]). In other symptom scales, between-group differences were not significant at 8 weeks (Table 3).

**Table 2:** Analyses of EORTC QLQ-C30 Global and functioning scales.

Scale (possible range)	Statistics	AMT (*N* = 20)	No-AMT (*N* = 20)	Difference (95% CI)^a^	*P*-value^b^
***Global Health Status / QOL*** (0–100)					
Baseline	Median (IQR)	75 (63 to 83)	79 (67 to 83)		
8-week follow-up	Median (IQR)	83 (67 to 88)	67 (50 to 83)	8.3 (0.0 to 16.7)	0.042
***Physical Functioning*** (0–100)					
Baseline	Median (IQR)	93 (87 to 100)	93 (87 to 100)		
8-week follow-up	Median (IQR)	93 (87 to 100)	93 (87 to 100)	0.0 (0.0 to 6.7)	0.755
***Role Functioning*** (0–100)					
Baseline	Median (IQR)	100 (100 to 100)	100 (100 to 100)		
8-week follow-up	Median (IQR)	100 (100 to 100)	100 (100 to 100)	0.0 (0.0 to 0.0)	0.919
***Emotional Functioning*** (0–100)					
Baseline	Median (IQR)	88 (71 to 92)	83 (67 to 96)		
8-week follow-up	Median (IQR)	79 (67 to 88)	79 (67 to 88)	0.0 (-8.3 to 8.3)	0.896
***Cognitive Functioning*** (0–100)					
Baseline	Median (IQR)	67 (67 to 83)	67 (50 to 83)		
8-week follow-up	Median (IQR)	83 (67 to 92)	83 (67 to 83)	0.0 (0.0 to 16.7)	0.618
***Social Functioning*** (0–100)					
Baseline	Median (IQR)	100 (100 to 100)	100 (100 to 100)		
8-week follow-up	Median (IQR)	100 (100 to 100)	100(100 to 100)	0.0 (NC to NC)	0.626

AMT = *Anma* therapy; CI = confidence interval; IQR = interquartile range; NC = not calculated for this problem with alpha = 0.05.

^a^Median differences between groups and their exact Hodges-Lehmann 95% confidence intervals of 8-week QOL differences from baselines were calculated.

^b^Exact *P*-values were calculated by Mann-Whitney U test.

**Table 3:** Analyses of EORTC QLQ-C30 symptom scales.

Scale (possible range)	Statistics	AMT (*N* = 20)	No-AMT (*N* = 20)	Difference (95% CI)^a^	*P*-value^b^
***Fatigue*** (0–100)					
Baseline	Median (IQR)	33 (22 to 44)	22 (11 to 33)		
8-week follow-up	Median (IQR)	28 (17 to 33)	33 (11 to 33)	-11.1 (-22.2 to 0.0)	0.047
***Nausea and vomiting*** (0–100)					
Baseline	Median (IQR)	0 (0 to 0)	0 (0 to 0)		
8-week follow-up	Median (IQR)	0 (0 to 0)	0 (0 to 0)	0.0 (NC to NC)	0.506
***Pain*** (0–100)					
Baseline	Median (IQR)	17 (0 to 33)	8 (0 to 17)		
8-week follow-up	Median (IQR)	17 (0 to 33)	17 (0 to 17)	0.0 (0.0 to 0.0)	0.682
***Dyspnea*** (0–100)					
Baseline	Median (IQR)	0 (0 to 17)	0 (0 to 17)		
8-week follow-up	Median (IQR)	0 (0 to 0)	0 (0 to 33)	0.0 (0.0 to 0.0)	0.277
***Insomnia*** (0–100)					
Baseline	Median (IQR)	33 (0 to 33)	0 (0 to 0)		
8-week follow-up	Median (IQR)	0 (0 to 33)	17 (0 to 33)	-33.3 (-33.3 to 0.0)	0.003
***Appetite loss*** (0–100)					
Baseline	Median (IQR)	0 (0 to 0)	0 (0 to 0)		
8-week follow-up	Median (IQR)	0 (0 to 0)	0 (0 to 0)	0.0 (0.0 to 0.0)	0.226
***Constipation*** (0–100)					
Baseline	Median (IQR)	33 (0 to 33)	0 (0 to 33)		
8-week follow-up	Median (IQR)	0 (0 to 33)	0 (0 to 33)	0.0 (0.0 to 0.0)	0.158
***Diarrhoea*** (0–100)					
Baseline	Median (IQR)	0 (0 to 0)	0 (0 to 17)		
8-week follow-up	Median (IQR)	0 (0 to 0)	0 (0 to 17)	0.0 (0.0 to 0.0)	1.000
***Financial difficulties*** (0–100)					
Baseline	Median (IQR)	0 (0 to 0)	0 (0 to 0)		
8-week follow-up	Median (IQR)	0 (0 to 0)	0 (0 to 0)	0.0 (NC to NC)	1.000

AMT = *Anma* therapy; CI = confidence interval; IQR = interquartile range; NC = not calculated for this problem with alpha = 0.05.

^a^Median differences between groups and their exact Hodges-Lehmann 95% confidence. intervals of 8-week QOL differences from baselines were calculated.

^b^Exact P-values were calculated by Mann-Whitney U test.

#### HADS

We found no significant between-group difference in scores for HADS anxiety (median scores, from 6 to 5 and from 5 to 6, respectively; *P* = 0.256) or HADS depression (median scores, from 4 to 3 and from 4 to 4, respectively; *P* = 0.282) over the 8-week trial (Table 4).

**Table 4:** Analyses of Hospital Anxiety Depression Scale (HADS) scale.

Scale (possible range)	Statistics	AMT (*N* = 20)	No-AMT (*N* = 20)	Difference (95% CI)^a^	*P*-value^b^
***HADS Anxiety*** (0–21)					
Baseline	Median (IQR)	6 (3 to 7)	5 (2 to 7)		
8-week follow-up	Median (IQR)	5 (2 to 7)	6 (3 to 8)	-1.0 (-2.0 to 1.0)	0.256
***HADS Depression*** (0–21)					
Baseline	Median (IQR)	4 (2 to 7)	4 (2 to 7)		
8-week follow-up	Median (IQR)	3 (2 to 5)	4 (3 to 7)	-1.0 (-3.0 to 1.0)	0.282

AMT = *Anma* therapy; CI = confidence interval; IQR = interquartile range.

^a^Median differences between groups and their exact Hodges-Lehmann 95% confidence intervals of 8-week QOL differences from baselines were calculated.

^b^Exact P-values were calculated with the use of Mann-Whitney U test.

#### POMS

Anger-hostility in the AMT group compared with the no-AMT group showed significant improvement at 8 weeks (median scores, from 2 to 1 and from 0 to 0, respectively; *P* = 0.028) (S1 Table). In other scales, between-group differences were not significant at 8 weeks. We found significant between-group differences in score changes from pre-session to post-session for anger-hostility (median scores, from 2 to 0 and from 0 to 0, respectively; *P* = 0.044), confusion (median scores, from 5 to 2 and from 4 to 4, respectively; *P* = 0.036), and total mood disturbance (median scores, from 5 to -8 and from 5 to 1, respectively; *P* = 0.028). Other scales showed no significant between-group differences (S1 Table).

#### MAC

No significant between-group differences were found in MAC score changes over the 8-week trial (S2 Table).

## Discussion

We have already confirmed that AMT significantly reduces the severity of physical complaints as assessed by VAS in survivors of gynecologic cancer [11]. In the present study, the Global Health Status/QOL was significantly improved in the AMT group and the estimated mean difference was clinically meaningful at 8 weeks. Also, scores on fatigue and insomnia were significantly improved in the AMT group and the estimated mean difference of insomnia was clinically meaningful. These findings suggest that reduced severity of physical complaints by AMT are accompanied by improved HR-QOL, especially by alleviating insomnia.

Although other studies have reported significant improvements in insomnia by massage therapies, some of these studies were based on different scales of insomnia, different massage therapies, or included subjects with various types or stages of cancer [25–27]. Some studies have reported that insomnia is one of the most prevalent physical symptoms in cancer survivors [1, 2, 4], which might support our hypothesis that AMT improved HR-QOL by alleviating insomnia.

Previous studies have indicated that cancer survivors experience anxiety [3–6] and depression [2–6]. However, in the present study, baseline medians of HADS anxiety and HADS depression in both AMT and no-AMT groups corresponded to the same categories as mentally healthy Japanese (cut-off points: 10 for anxiety and 5 for depression) [28]. This might account for our inability to confirm a reduction in participants’ anxiety and depression after AMT.

Several recent studies have reported improved mood after massage in patients with cancer, although the method used in some studies to assess and confirm this was based on different scales, different massage therapies, or included subjects with various stages of cancer [29–33]. Our results are very similar to those reported in previous studies using POMS assessment: significant improvement in anger-hostility and total mood disturbance after massage [34]; immediate and significant decreased mood disturbance compared to a control group [35]; and less anger-hostility after longer-term massage [36]. Moreover, a meta-analysis including 18 RCTs (950 breast cancer patients in total) revealed no significant improvement in depression and anxiety, although it did find significant improvement in anger by massage compared with control interventions [37]. The psychological/emotional results of the present study clearly support our findings.

For patients with cancer, coping with the disease is an important determinant of psychological morbidity, QOL, and treatment adherence [38]. Poor coping responses (i.e., low fighting spirit, high anxious preoccupation, high fatalism, and high helplessness/hopelessness) were found to be significant determinants of psychiatric morbidity in ambulatory breast cancer patients [39]. Patients with ovarian cancer who used massage therapy were found to have significantly lower hopelessness [40]. However, in this study, we found no significant differences in any MAC scale score over the 8-week trial period. Further studies are needed to investigate this point.

This study has some limitations. First, participants might have been psychologically healthy and without serious symptoms such as nausea and vomiting, dyspnea, appetite loss, or financial difficulty as measured by the EORTC QLQ-C30 (Tables 2 and 3). It could be that they were comparatively long-term survivors without recurrence at the time of the trial. It is also possible that questionnaires such as the HADS, MAC, and EORTC QLQ-C30—each developed specifically to target patients with cancer—were not suitable to verify the effects of AMT on the mindset and HR-QOL of the present participants.

Second, as this was the first registered RCT on the effects of AMT and given the absence of basic scientific data, medical doctors had difficulty recruiting patients with cancer currently receiving medical treatment. Third, to generalize findings from this RCT, findings should be verified by a multicenter RCT that utilizes carefully selected questionnaires, recruits a more consistent population, and limits sample population characteristics (e.g., by tumor type, cancer stage, duration from cancer onset, or target symptoms). Fourth, a precise no-AMT intervention (control) might entail equal division between the two groups of the total amount of time spent with a therapist. A subsequent RCT should reconsider the appropriate intervention method for the control for AMT. Non-*Anma* relaxation practices such as yoga or meditation might be a better intervention for the control group. Finally, this study was an open-label RCT and subjective assessments were needed for most endpoints, which might lead to biased results. For greater credibility of our findings, we need a double-blinded (to the extent possible) multicenter RCT with better active control intervention in the future.

In conclusion, as well as reducing the severity of physical complaints [11], continuous once-weekly AMT over a period of 8 weeks improved the HR-QOL of gynecologic cancer survivors. It is noteworthy that our findings are very similar to those of Western clinical studies on massage, and implies that Japanese massage (AMT) has benefits in oncology similar to those of common massage techniques reported in Western studies.

## Supporting information

S1 Checklist CONSORT checklist. (DOC)

S1 Fig CONSORT flow diagram. (DOC)

S1 Table Analyses of Profile of Mood States (POMS) scales. AMT = *Anma* therapy; CI = confidence interval; IQR = interquartile range.
^a^Median differences between groups and their exact Hodges-Lehmann 95% confidence intervals of post-session or 8-week QOL differences from baselines were calculated.
^b^Exact *P*-values were calculated by Mann-Whitney U test.
^c^Exact *P*-values were estimated by Monte Carlo estimation (1,000,000 samples).
(DOCX)

S2 Table Analyses of Measure of Adjustment to Cancer (MAC) scales. AMT = *Anma* therapy; CI = confidence interval; IQR = interquartile range.
^a^Median differences between groups and their exact Hodges-Lehmann 95% confidence intervals of 8-week QOL differences from baselines were calculated.
^b^Exact *P*-values were calculated by Mann-Whitney U test.
(DOCX)

S1 Dataset This dataset contains the full data for participant characteristics, EORTC QLQ-C30, HADS-anxiety, HADS-depression, POMS, and MAC. (ZIP)

S1 Protocol This document contains the study protocol, which is written in Japanese. Although the trial has not been translated into English, some information is available in English from the UMIN Clinical Trials Registry (https://upload.umin.ac.jp/cgi-open-bin/ctr/ctr.cgi?function=brows&action=brows&type=summary&recptno=R000010670&language=E).
(DOC)
