# Supplementary material for: Effects of Anma therapy (Japanese massage) on health-related quality of life in gynecologic cancer survivors: A randomized controlled trial
Source: PLoS One. 2018 May 3;13(5):e0196638. doi: 10.1371/journal.pone.0196638 (PMC5933696; doi:10.1371/journal.pone.0196638)
Supplement: S1 Fig — (DOC) [file pone.0196638.s002.doc]

**
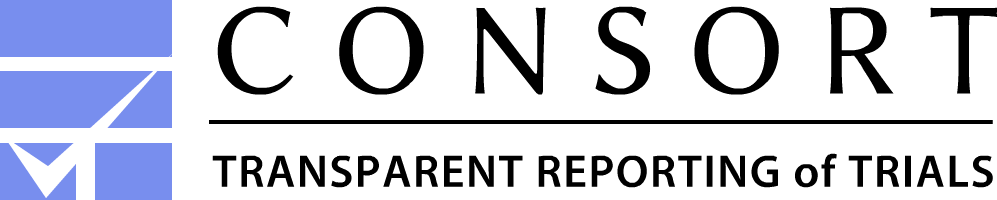
**

**CONSORT 2010 Flow Diagram**

**Allocation**

**Analysis**

**Follow-Up**

**Enrollment**

Assessed for eligibility (n=58)

Excluded (n=18)

♦  Not meeting inclusion criteria (n=0)

♦  Declined to participate (n=18)

♦  Other reasons (n=0)

Analyzed (n=20)
♦ Excluded from analysis (n=0)

Lost to follow-up (n=0)

Discontinued intervention (n=0)

Allocated to intervention (n=20)

♦ Received allocated intervention (n=20)

♦ Did not receive allocated intervention (n=0)

Lost to follow-up (n=0)

Discontinued intervention (n=0)

Allocated to intervention (n=20)

♦ Received allocated intervention (n=19)

♦ Did not receive allocated intervention (according to the mistake, a patient was treated as a member of another group) (n=1)

Analyzed (n=20)
♦ Excluded from analysis (n=0)

Randomized (n=40)
