# Supplementary material for: Effects of Anma therapy (Japanese massage) on health-related quality of life in gynecologic cancer survivors: A randomized controlled trial
Source: PLoS One. 2018 May 3;13(5):e0196638. doi: 10.1371/journal.pone.0196638 (PMC5933696; doi:10.1371/journal.pone.0196638)
Supplement: S1 Table — AMT = Anma therapy; CI = confidence interval; IQR = interquartile range. aMedian differences between groups and their exact Hodges-Lehmann 95% confidence intervals of post-session or 8-week QOL differences from baselines were calculated. bExact P-values were calculated by Mann-Whitney U test. cExact P-values were estimated by Monte Carlo estimation (1,000,000 samples). (DOCX) [file pone.0196638.s003.docx]

S1 Table. Analyses of Profile of Mood States (POMS) Scales

| **Scale (possible range)** | **Statistics** | **AMT (*N* = 20)** | **No-AMT (*N* = 20)** | **Difference (95% CI)**^a^ | ***P*-value**^b^ |
| --- | --- | --- | --- | --- | --- |
| ***Tension-Anxiety*** (0–20) | | | | | |
| Pre-session | Median (IQR) | 3 (2 to 6) | 2 (1 to 4) |  |  |
| Post-session | Median (IQR) | 0 (0 to 1) | 0 (0 to 2) | -1.0 (-3.0 to 1.0) | 0.245 |
| 8-week follow-up | Median (IQR) | 2 (0 to 5) | 2 (1 to 3) | -1.0 (-3.0 to 1.0) | 0.540 |
| ***Depression-Dejection*** (0–20) | | | | | |
| Pre-session | Median (IQR) | 2 (0 to 3) | 1 (0 to 2) |  |  |
| Post-session | Median (IQR) | 0 (0 to 1) | 0 (0 to 1) | -1.0 (-2.0 to 0.0) | 0.184 |
| 8-week follow-up | Median (IQR) | 1 (1 to 3) | 1 (0 to 3) | 0.0 (-1.0 to 2.0) | 0.478 |
| ***Anger-Hostility*** (0–20) | | | | | |
| Pre-session | Median (IQR) | 2 (1 to 5) | 0 (0 to 1) |  |  |
| Post-session | Median (IQR) | 0 (0 to 0) | 0 (0 to 0) | -1.0 (-2.0 to 0.0) | 0.044 |
| 8-week follow-up | Median (IQR) | 1 (0 to 4) | 0 (0 to 2) | -1.0 (-2.0 to 0.0) | 0.028 |
| ***Vigor*** (0–20) | | | | | |
| Pre-session | Median (IQR) | 8 (7 to 11) | 7 (3 to 10) |  |  |
| Post-session | Median (IQR) | 10 (6 to 14) | 7 (3 to 10) | 1.0 (-1.0 to 4.0) | 0.328 |
| 8-week follow-up | Median (IQR) | 10 (7 to 14) | 6 (3 to 8) | 2.0 (-1.0 to 5.0) | 0.197 |
| ***Fatigue*** (0–20) | | | | | |
| Pre-session | Median (IQR) | 3 (0 to 4) | 1 (0 to 3) |  |  |
| Post-session | Median (IQR) | 0 (0 to 1) | 0 (0 to 1) | -1.0 (-3.0 to 0.0) | 0.050 |
| 8-week follow-up | Median (IQR) | 2 (1 to 6) | 1 (0 to 5) | -1.0 (-2.0 to 0.0) | 0.113 |
| ***Confusion*** (0–20) | | | | | |
| Pre-session | Median (IQR) | 5 (4 to 7) | 4 (4 to 6) |  |  |
| Post-session | Median (IQR) | 2 (2 to 4) | 4 (2 to 4) | -2.0 (-3.0 to 0.0) | 0.036 |
| 8-week follow-up | Median (IQR) | 4 (3 to 6) | 4 (3 to 6) | -1.0 (-3.0 to 1.0) | 0.262 |
| ***Total Mood Disturbance*** (-20–100) | | | | | |
| Pre-session | Median (IQR) | 5 (-2 to 16) | 5 (-1 to 11) |  |  |
| Post-session | Median (IQR) | -8 (-11 to 2) | 1 (-6 to 6) | -6.0 (-14.0 to -1.0) | 0.028^c^ |
| 8-week follow-up | Median (IQR) | 2 (-5 to 11) | 4 (-3 to 14) | -4.0 (-12.0 to 3.0) | 0.249^c^ |

AMT = *Anma* therapy; CI = confidence interval; IQR = interquartile range.

^a^Median differences between groups and their exact Hodges-Lehmann 95% confidence intervals of post-session or 8-week QOL differences from baselines were calculated.

^b^Exact *P*-values were calculated by Mann-Whitney U test.

^c^Exact *P*-values were estimated by Monte Carlo estimation (1,000,000 samples).
