# Supplementary material for: Effects of Anma therapy (Japanese massage) on health-related quality of life in gynecologic cancer survivors: A randomized controlled trial
Source: PLoS One. 2018 May 3;13(5):e0196638. doi: 10.1371/journal.pone.0196638 (PMC5933696; doi:10.1371/journal.pone.0196638)
Supplement: S2 Table — AMT = Anma therapy; CI = confidence interval; IQR = interquartile range. aMedian differences between groups and their exact Hodges-Lehmann 95% confidence intervals of 8-week QOL differences from baselines were calculated. bExact P-values were calculated by Mann-Whitney U test. (DOCX) [file pone.0196638.s004.docx]

S2 Table. Analyses of Measure of Adjustment to Cancer (MAC) Scales

| **Scale (possible range)** | **Statistics** | **AMT (*N* = 20)** | **No-AMT (*N* = 20)** | **Difference (95% CI)**^a^ | ***P*-value**^b^ |
| --- | --- | --- | --- | --- | --- |
| ***Fighting spirit*** (16–64) | | | | | |
| Baseline | Median (IQR) | 49 (42 to 54) | 48 (45 to 53) |  |  |
| 8-week follow-up | Median (IQR) | 52 (44 to 56) | 47 (42 to 53) | 3.0 (0.0 to 7.0) | 0.054 |
| ***Helplessness/hopelessness*** (6–24) | | | | | |
| Baseline | Median (IQR) | 8 (7 to 10) | 9 (7 to 10) |  |  |
| 8-week follow-up | Median (IQR) | 8 (6 to 9) | 7 (6 to 9) | 0.0 (-1.0 to 1.0) | 0.769 |
| ***Anxious preoccupation*** (9–36) | | | | | |
| Baseline | Median (IQR) | 19 (17 to 22) | 18 (15 to 23) |  |  |
| 8-week follow-up | Median (IQR) | 19 (17 to 21) | 17 (15 to 20) | 0.0 (-2.0 to 2.0) | 0.740 |
| ***Fatalism*** (8–32) | | | | | |
| Baseline | Median (IQR) | 18 (15 to 20) | 20 (17 to 23) |  |  |
| 8-week follow-up | Median (IQR) | 17 (14 to 21) | 18 (17 to 21) | 1.0 (-1.0 to 3.0) | 0.367 |
| ***Avoidance*** (1–4) | | | | | |
| Baseline | Median (IQR) | 1 (1 to 2) | 2 (2 to 3) |  |  |
| 8-week follow-up | Median (IQR) | 1 (1 to 3) | 2 (1 to 3) | 0.0 (-1.0 to 1.0) | 0.632 |

AMT = *Anma* therapy; CI = confidence interval; IQR = interquartile range.

^a^Median differences between groups and their exact Hodges-Lehmann 95% confidence intervals of 8-week QOL differences from baselines were calculated.

^b^Exact *P*-values were calculated by Mann-Whitney U test.
