# Supplementary material for: Effects of Anma therapy (Japanese massage) on health-related quality of life in gynecologic cancer survivors: A randomized controlled trial
Source: PLoS One. 2018 May 3;13(5):e0196638. doi: 10.1371/journal.pone.0196638 (PMC5933696; doi:10.1371/journal.pone.0196638)
Supplement: S1 Protocol — Although the trial has not been translated into English, some information is available in English from the UMIN Clinical Trials Registry (https://upload.umin.ac.jp/cgi-open-bin/ctr/ctr.cgi?function=brows&action=brows&type=summary&recptno=R000010670&language=E). (DOC) [file pone.0196638.s006.doc]

文部科学省科学研究費補助金平成22～26年度基盤Ｃ

『按摩マッサージ療法のエビデンスに基づいた職域の開拓：緩和ケアチームへの参入』

（課題番号22531058 研究代表者：殿山　希）

**『がんサバイバーに対する継続的按摩療法の効果に関する研究（ランダム化比較試験）』**

**実施計画書 ver0.1**

研究代表者： 殿山　希　　筑波技術大学保健科学部保健学科鍼灸学専攻

〒305-8521　茨城県つくば市春日4-12-7

TEL & FAX： 029-858-9631

E-mail： donoyama@k.tsukuba-tech.ac.jp

研究事務局： 殿山　希　　筑波技術大学保健科学部保健学科鍼灸学専攻

〒305-8521　茨城県つくば市春日4-12-7

TEL & FAX： 029-858-9631

E-mail： donoyama@k.tsukuba-tech.ac.jp

2012年XX月XX日　筑波技術大学医の倫理委員会プロトコールコンセプト承認

1. 概要

0.1. シェーマ

他施設医療機関

①研究対象のリクルート

（対象基準に合った患者へ研究参加の情報提供・研究事務局紹介）

筑波技術大学

②対象への説明

③同意書提出＝登録

④対象の割り付け：按摩継続群・按摩1回群

測定C・測定E・測定F・測定H：血液、尿、VAS、唾液、質問紙

測定A・B・D・G・I：VAS、唾液

【評価】

1.継続的按摩療法の効果

測定C－－測定E 按摩継続の効果（按摩継続群）

測定F－－測定H　コントロール（按摩１回群）

2.　按摩施術の直後効果（副次的評価項目）

測定C－－測定D、測定H－－測定I 直後効果 VS 測定A－－測定B、測定F－－測定G コントロール

0.2. 目的

本研究の目的は、がんサバイバーに対する継続的按摩療法の有効性を検証することである。

Primary endpointは自覚症状の変化（Visual Analogue Scaleによる）

Secondary endpointsは下記の通りである。

Hospital Anxiety Depression Scale (HADS)スコア

European Organization for Research and Treatment of Cancer QLQ-C30 (EORTC QLQ-C30) スコア

日本語版MACスコア

白血球分画

Natual Killer cell numbers・activity

リンパ球（Th1, Th2, 数と比）

IL-6

高感度CRP

尿中酸化ストレス物質（8-hydroxydeoxyguanosine[8-OH-dG]他)

Salivary cortisol

Chromogranin A

s-immunoglobulin A (s-IgA)

0.3. 対象

本試験の対象はがんサバイバーである。以下の適格規準をすべて満たし、除外規準のいずれにも該当しない患者を登録適格例とする。

適格規準

1. 過去に組織学的に子宮頸部、子宮体部、卵巣・卵管・腹膜がんが確認された患者。
2. 標準的治療終了後3年以上を経過し臨床的に再発の兆候がない患者。
3. 登録時に十分なインフォームドコンセントに基づき患者が按摩療法を希望している。
4. 登録時の年齢が20歳以上。
5. 主治医が本試験に登録可能と判断できる。
6. 登録時に試験参加について、本人より文書による同意(インフォームド・コンセント)が得られている。

除外規準

　1） 活動性の感染症がない。

　2）心臓・肝臓・腎臓機能などに治療を要する障害がない。

　3）認知に問題がない。

0.4. 治療

本試験は、按摩療法のがん患者への有効性を検証するものである。そこで、

がんサバイバーに対して、実験的介入研究を行い、按摩療法8週介入群と非介入群を比較する。また、副次的評価として、按摩1回施術による直後効果を確認する。

　対象は毎週1回40分程度の按摩施術を8週間、合計8回継続的に受ける。

　按摩施術は日本で通常行われている手技により衣服の上から全身に行う。手順は資料1に従う。なお、特に改善を希望する自覚的な症状の部位やその緩和に関連する部位に対しては綿密に施術を行う。施術強度は対象が「心地よい」と感じる強さとする。

　施術者の技術力による結果のばらつきを防ぐために施術は毎回原則として同一施術者が行う。身体的愁訴の改善に力点を置いた施術とする。

0.5. 予定登録数と研究期間

予定登録被験者数：60名

研究期間：2年

0.6. 問い合わせ先

殿山　希　　筑波技術大学保健科学部保健学科鍼灸学専攻

〒305-8521　茨城県つくば市春日4-12-7

TEL & FAX： 029-858-9631

E-mail： donoyama@k.tsukuba-tech.ac.jp

目次

1. 目的　・・・・・・・・・・・・・・・・・・・・・・・・・・・・・・・・6

1.1. 目的　・・・・・・・・・・・・・・・・・・・・・・・・・・・・・・・・6

1.2. Research Questions　・・・・・・・・・・・・・・・・・・・・・・・・・6

2. 背景と試験計画の根拠　・・・・・・・・・・・・・・・・・・・・・・・・6

2.1. 背景　・・・・・・・・・・・・・・・・・・・・・・・・・・・・・・・・6

2.2． 試験計画の根拠　・・・・・・・・・・・・・・・・・・・・・・・・・・・7

2.3. 本試験の意義　・・・・・・・・・・・・・・・・・・・・・・・・・・・・8

3. 本試験で用いる規準・定義　・・・・・・・・・・・・・・・・・・・・・・8

4. 患者選択規準　・・・・・・・・・・・・・・・・・・・・・・・・・・・・8

5. 登録・割付　・・・・・・・・・・・・・・・・・・・・・・・・・・・・・9

5.1. 登録の手順　・・・・・・・・・・・・・・・・・・・・・・・・・・・・・9

5.1.1.登録に際しての注意事項　・・・・・・・・・・・・・・・・・・・・・・・9

5.2. ランダム割付と割付調整因子　・・・・・・・・・・・・・・・・・・・・・9

6. 治療計画と治療変更規準　・・・・・・・・・・・・・・・・・・・・・・・10

6.1. プロトコール治療　・・・・・・・・・・・・・・・・・・・・・・・・・・10

6.2. プロトコール治療中止・完了規準　・・・・・・・・・・・・・・・・・・・10

6.3. 治療変更規準　・・・・・・・・・・・・・・・・・・・・・・・・・・・・10

6.4. 併用療法・支持療法　・・・・・・・・・・・・・・・・・・・・・・・・・11

6.5. 後治療　・・・・・・・・・・・・・・・・・・・・・・・・・・・・・・・11

7. 予期される有害反応　・・・・・・・・・・・・・・・・・・・・・・・・・11

8. 評価項目・評価スケジュール　・・・・・・・・・・・・・・・・・・・・・11

8.1. 評価項目とその設定根拠　・・・・・・・・・・・・・・・・・・・・・・・11

8.2. 評価スケジュール　・・・・・・・・・・・・・・・・・・・・・・・・・・12

9. データ収集と管理　・・・・・・・・・・・・・・・・・・・・・・・・・・12

10. 有害事象の報告　・・・・・・・・・・・・・・・・・・・・・・・・・・・13

11. 解析対象集団の定義　・・・・・・・・・・・・・・・・・・・・・・・・・13

11.1. 解析対象集団の定義　・・・・・・・・・・・・・　・・・・・・・・・・・13

11.1.1.全登録例　・・・・・・・・・・・・・・・・・・・・・・・・・・・・・・13

11.1.2.全適格例　・・・・・・・・・・・・・・・・・・・・・・・・・・・・・・13

11.1.3.全治療例　・・・・・・・・・・・・・・・・・・・・・・・・・・・・・・13

12. 統計学的事項　・・・・・・・・・・・・・・・・・・・・・・・・・・・・13

12.1. 主たる解析と判断規準　・・・・・・・・・・・・・・・・・・・・・・・・13

12.2. 症例数設定の根拠　・・・・・・・・・・・・・・・・・・・・・・・・・・14

12.3. Secondary endpointsの解析　・・・・・・・・・・・・・・・・・・・・・15

12.4. その他の事項　・・・・・・・・・・・・・・・・・・・・・・・・・・・・15

13. 倫理的事項　・・・・・・・・・・・・・・・・・・・・・・・・・・・・・15

13.1. 患者の保護　・・・・・・・・・・・・・・・・・・・・・・・・・・・・・15

13.2. インフォームドコンセント　・・・・・・・・・・・・・・・・・・・・・・15

13.2.1.患者への説明　・・・・・・・・・・・・・・・・・・・・・・・・・・・・15

13.2.2.同意　・・・・・・・・・・・・・・・・・・・・・・・・・・・・・・・・16

13.3.プロトコールの内容変更について　・・・・・・・・・・・・・・・・・・・・16

13.4.補償および患者の経済的負担について　・・・・・・・・・・・・・・・・・・16

13.4.1.補償について　・・・・・・・・・・・・・・・・・・・・・・・・・・・・16

13.4.2.患者の経済的負担について　・・・・・・・・・・・・・・・・・・・・・・16

13.4.3.研究代表者　・・・・・・・・・・・・・・・・・・・・・・・・・・・・・16

文献　　 ・・・・・・・・・・・・・・・・・・・・・・・・・・・・・・・・・・17

資料１　 ・・・・・・・・・・・・・・・・・・・・・・・・・・・・・・・・・・20

1. 目的

1.1. 目的

　本研究の目的は、がんサバイバーに対する継続的按摩療法の有効性を検証することである。

Primary endpointは自覚症状の変化（Visual Analogue Scaleによる）

Secondary endpointsは下記の通りである。

・Hospital Anxiety Depression Scale (HADS)スコア

・European Organization for Research and Treatment of Cancer QLQ-C30 (EORTC QLQ-C30) スコア

・日本語版MACスコア

・白血球分画

・Natual Killer cell numbers・activity

・リンパ球（Th1, Th2, 数と比）

・IL-6

・高感度CRP

・尿中酸化ストレス物質（8-hydroxydeoxyguanosine[8-OH-dG]他)

・Salivary cortisol

・Chromogranin A

・s-immunoglobulin A (s-IgA)

1.2. Research Questions

継続的按摩療法について以下のResearch Questionsを設ける。

　按摩施術を継続することでがんサバイバーにとって

①がん治療後に現れた身体的な愁訴を軽減できるか？

②精神的な愁訴を軽減できるか？

③健康関連QOLを高めることができるか？

2. 背景と試験計画の根拠

2.1.　背景

　按摩療法は、施術者の手で被術者の体表に軽擦法（触れ、撫で、擦する）、揉捏法（揉み捏ねる）、圧迫法（圧す）などの手技を加えて触圧覚刺激を行う日本古来から伝わる手技療法である。

　按摩は『導引按』として６世紀に中国から伝わった。導引は筋や関節の運動法と呼吸法であり、按は手で行う療法、いわゆる按摩法である。古来の按摩は経脈の流れの状態により五臓六腑を候い、経絡に沿って虚実に応じて補瀉を行い、気血の流れを整え、病気の予防や治療を行うという東洋医学理論に基づいていた。江戸時代には按摩の専門書も多く書かれて当時の医学に取り込まれ、日本の文化や人々の嗜好に合わせて発展した。

　一方、西洋医学理論に基づくヨーロッパのマッサージの本が1885年に軍医によりヨーロッパから伝えられ、1891年に『按摩術』という名で翻訳された。^1)^明治時代には多くのマッサージ書が発刊され、この頃に日本では東西の手技療法である按摩とマッサージの技術や理論は融合され、今日「按摩療法」「按摩マッサージ」と呼ばれる日本の手技療法の原形がつくられた。現在、日本では按摩療法は現代医学の解剖学に基づいて施術され、生理学的に解釈される。人々からもっとも親しまれている補完代替医療のひとつとして健康の維持・増進、疾病によりもたらされる症状の軽減・緩和、疾病の予防などに用いられている。

2.2．試験計画の根拠

　米国のマッサージ研究所 Touch Research Instituteでは、独自で行った100以上の研究からマッサージの効果として、未熟児の体重増加、記銘力の向上、鎮痛、抑うつの緩和、ストレスホルモンの低下、免疫機能の賦活を挙げている。^2)^また、同様の効果について多くの研究者が支持している。

　按摩療法の効果としては、古くより興奮作用、鎮静作用、反射作用、誘導作用、矯正作用が言われているが、^3)^科学的エビデンスを示した研究は少ない。

　東洋医学は心身一如の医学であることから、著者らは按摩療法の「身体」と「心理」への影響を研究してきた。

(1) 健康成人に対する効果

　慢性肩こりを訴える健康女性に対して40分間の按摩マッサージを行い、直後効果として①visual analogue scale (VAS)で測定した肩こりの自覚症状の軽減、②STAI^4)^で測定した不安の低下、③ストレスホルモン（唾液中コルチゾル）の低下、④免疫機能の賦活（分泌型IgAの増加）を確認した。^5)^

　按摩施術は体表から機械的刺激を与える。体性感覚神経から入力した刺激は脳へと伝わり、免疫系への反応や自律神経系への影響など、全身にさまざまな反応をもたらすことが考えられる。また、精神や心理への影響も考えられる。

　そこで、次に、按摩療法の臨床効果にどのようなメカニズムが関与するのかを検討するために施術により発現変動する遺伝子パターンを検討した。健康女性4人に40分間の全身按摩施術を行い、その前後に採血を行い、白血球中のRNAを抽出して41,000の全遺伝子発現統計解析を行った。施術によって免疫機能に関わる遺伝子や代謝系に関わる遺伝子が多く発現変動していたことから按摩マッサージは免疫系疾患や代謝系疾患の治療や予防に貢献することが示唆された。^6)^

　これらの先行研究から按摩療法は健康成人のみならず、補完代替医療として、心身に不調をかかえる人に対しても貢献できることが考察された。

(2) がんサバイバーに対する按摩療法の効果

　厚生労働省がん研究助成金による「地域がん登録」研究班によると、2020年には日本のがん罹患者数は85万人に達すると推計されている。^7)^2010年には男性がん患者数はピークに達してその後減少するが、女性がん患者数、特に、子宮がん、乳がん、結腸がんはその後も増加することが推測されている。^8)^しかし、これらのがんは、早期で発見、治療すれば、比較的予後のよいがんであるとされ、短期、および長期生存者数の推定においても上位を占めており、^9)^これは、治療を受け生存する「がんサバイバー」とよばれる患者が増加していくことを示している。がんサバイバーは、治療終了後も再発や死への不安を恒常的に抱えており、心身ともに非常にストレスフルな状況にあることが知られている。^10-12)^

　マッサージががん患者に有効であるという報告がある。^13-15)^また、Billhult and Dahl-berg^16)^はマッサージががん患者の心身の愁訴の軽減に貢献することを報告している。実際、マッサージ療法はがん患者がもっとも多く利用している代替医療のひとつである。^17)^そこで、私たちはがんサバイバーの心身に按摩療法は有用であるかを探索するために予備研究を行った。

　子宮がん摘出後２年以内で予後良好と考えられる５人のがん生存者に対して40分間の按摩施術を週に２回ずつ４週間行った。その結果、按摩施術の直後効果として、健康者と同様、身体的な自覚症状の有意な改善、状態不安の低下、ストレスホルモンの低下、免疫機能の指標である分泌型IgAの有意な増加が認められた。^18)^先行研究では、乳がん患者に対してマッサージを行った後、NK細胞やリンパ球数が増加したという報告もあり、^19)^手技療法の免疫機能への関与が考えられる。

また、4週間の按摩継続後では、自覚症状の継続的な軽減、不安感と抑うつの低下がみられた。^18)^がんサバイバー^12)^や担がん患者^10,11)^は多くの場合、不安や抑うつを感じており、その心理的状態は身体的な自覚症状と関係が深い。^20)^先行研究^19,21,22)^では、マッサージ施術は身体的・心理的愁訴からがんとともに生きる人を解放すると考察している。按摩療法により、心身の不調を軽減させることができれば、がんサバイバーや担がん患者の生活の質を向上させることにつながると考える。

　これらの先行研究を踏まえて、今回は十分なサンプル数を集めて按摩療法の心身への効果を明らかにする。

2.3. 本試験の意義

　本試験結果がpositiveであっても、negativeであっても現時点でcontroversialである按摩療法のがん患者への効果に対する解答となり、ガイドラインに反映されることが期待できる。また、今までは患者の意思のみで按摩療法が選択されてきたが、按摩療法を選択した場合のメリットをエビデンスを持って患者に説明でき、患者がより正しい情報に基づいて意思決定が出来る様になる。

3. 本試験で用いる規準・定義

特になし。

4. 患者選択規準

本試験の対象はがんサバイバーである。以下の適格規準をすべて満たし、除外規準のいずれにも該当しない患者を登録適格例とする。

適格規準

1. 過去に組織学的に子宮頸部、子宮体部、卵巣・卵管・腹膜がんが確認された患者。
2. 標準的治療終了後3年以上を経過し臨床的に再発の兆候がない患者。
3. 録時に十分なインフォームドコンセントに基づき患者が按摩療法を希望している。
4. 登録時の年齢が20歳以上。
5. 主治医が本試験に登録可能と判断できる。
6. 登録時に試験参加について、本人より文書による同意(インフォームド・コンセント)が得られている。

除外規準

1. 活動性の感染症がない。
2. 心臓・肝臓・腎臓機能などに治療を要する障害がない。
3. 認知に問題がない。

5. 登録・割付

5.1. 登録の手順

　適格規準をすべて満たし、除外規準のいずれにも該当しないことを確認した患者に研究事務局を紹介する。同時に、対象の担当医師は必要事項をすべて記入した登録適格性確認票を研究事務局にFAXする。対象は研究事務局に電話かメールで連絡して面談日を定め、事務局に訪れ、研究についての説明を口頭と書面により受ける。その後、研究に登録する場合は事務局に直接、あるいはFAXか郵送で同意書を提出する。

患者登録の連絡先

研究事務局： 殿山　希　　筑波技術大学保健科学部保健学科鍼灸学専攻

〒305-8521　茨城県つくば市春日4-12-7

TEL & FAX： 029-858-9631

E-mail： donoyama@k.tsukuba-tech.ac.jp

5.1.1. 登録に際しての注意事項

1) 登録適格性確認票の内容確認が不十分な時は、すべて満たされるまで登録は受付けられない。

2) データの研究利用の拒否を含む同意撤回があった場合を除いて、一度登録された患者は登録取り消しはなされない。

5.2. ランダム割付と割付調整因子

登録にあたって治療群は研究事務局でランダムに割り付けられる。ランダム割付の方法はランダム化ブロック法を用いる。現時点で予後因子に関する十分なエビデンスが存在しないことから、本試験では割付調整因子は設定しない。ランダム割付の方法に関する詳細は研究事務局の内規とし、記録は研究事務局で保管する。

6. 治療計画と治療変更規準

　患者の安全が脅かされない限りにおいて、治療および治療変更は本章の記述に従って行う。プロトコールに従えば医学的に危険と判断される場合は施術担当者の医学的判断に従って治療変更を行う。

6.1. プロトコール治療

　本試験は、按摩療法のがんサバイバーへの有効性を検証するものである。そこで、がんサバイバーに対して、実験的介入研究を按摩療法8週介入群と非介入群を比較する。

　対象は毎週1回40分程度の按摩施術を8週間、合計8回継続的に受ける。

按摩施術は日本で通常行われている手技により衣服の上から全身に行う。手順は資料1に従う。なお、特に改善を希望する自覚的な症状の部位やその緩和に関連する部位に対しては綿密に施術を行う。施術強度は対象が「心地よい」と感じる強さとする。

　施術者の技術力による結果のばらつきを防ぐために施術は毎回原則として同一施術者が行う。身体的愁訴の改善に力点を置いた施術とする。

6.2. プロトコール治療中止・完了規準

毎週1回40分程度の按摩施術を8週間、合計8回継続的に受けたときに、プロトコール治療完了とする。以下のいずれかの場合はプロトコール治療を中止する。

1. 増悪/再発：現病の増悪・再発が認められた場合
2. 有害事象：有害事象でプロトコール治療が継続できないと担当施術者、あるいは共同研究医師が判断した場合
3. 拒否（有害事象）：有害事象との関連が否定できない理由により、患者がプロトコール治療の中止を申し出た場合
4. 拒否（その他）：有害事象との関連が否定できる理由により、患者がプロトコール治療の中止を申し出た場合
5. 死亡：プロトコール治療中（観察期間も含む）の死亡（治療との関連を問わない）
6. その他：その他の理由による治療中止。登録後治療開始前の増悪、プロトコール違反、登録後の診断変更により不適格性が判明した場合

6.3. 治療変更規準

　以下、変更規準については次の用語を用いる。

　　中止：治療の一部または全部の、再開しない途中終了

　　休止：条件を満たせば再開する可能性のある一時的中断や休止

6.4. 併用療法・支持療法

　本試験開始後に新規にいずれの併用療法・支持療法も受けない。研究開始前から継続している療法がある場合は事前に知らせる。

6.5. 後治療

プロトコール治療中止後および完了後の治療は規定しない。

7. 予期される有害反応

　本プロトコール治療によって出現する特異的な有害事象の可能性はきわめて低いと考えられるが、按摩療法の反応として、施術後一過性の気分不良が生じる可能性、刺激量過剰による筋のこわばり・痛み（いわゆる「揉み返し」）が出現する場合もたまにある。時間が経過すれば回復するもので心配はないが、希望があれば医師による診察を行う。

8. 評価項目・評価スケジュール

8.1. 評価項目とその設定根拠

　本研究の評価項目を下記の通り定める。

主要評価項目

・自覚症状（Visual Analogue Scaleによる）

主要評価項目の設定根拠

被験者の自覚症状を評価する最も一般的な尺度であるため。Donoyama et. al.(2011)^18)^の予備研究においても主たる評価項目として設定・評価されている。

副次的評価項目

1. Hospital Anxiety and Depression Scale HADS)スコア

2. European Organization for Research and Treatment of Cancer QLQ-C30 (EORTC QLQ-C30) スコア

3. 日本語版MACスコア

4. salivary cortisol

5. chromogranin A

6. s-immunoglobulin A (s-IgA)

7. 白血球分画

8. Natual Killer cell numbers・activity

9. リンパ球（Th1, Th2, 数と比）

10. IL6

11. 高感度CRP

12. 尿中酸化ストレス物質（8-hydroxydeoxyguanosine[8-OH-dG]他)

副次的評価項目の設定根拠

　がんサバイバーやがん患者は治療終了後も再発や死への不安を恒常的に抱えており、心身ともに非常にストレスフルな状況にあることが知られている^10-12)^が、マッサージががん患者の不安や抑うつに効果がみられるという報告^16,18)^もある。そこで、がん患者向けに開発された不安・抑うつ尺度であるHospital Anxiety and Depression Scale (HADS)^23)^の日本語版尺度^24)^を用いて不安・抑うつを測定する。本尺度は既に信頼性・妥当性が確認されている。^25)^項目数が14と少ないことから対象への負担も少なく不安と抑うつを測定できる。

　また、マッサージによりがん患者のQuality of life (QOL)の向上も報告されていることから^26)^がん患者のQOL評価 として信頼性・妥当性が検証されているEORTC QLQ-C30尺度^27,28)^を用いてQOLを測定する。

コーピングは、患者の心理状態を決定づける先行要因である^29)^。がんに対するコーピングとQOLやメンタルヘルスとの関連についての研究からFighting spirits (闘病意欲)が高いほど良好なQOLが保てる一方、helplessness（無力感）やhopelessness(絶望)が高いほど、QOLは低下するということが明らかになっている^30,31)^。按摩施術を受け続けることで患者のコーピングに何らかの変化が生じることも考えられることからがんサバイバーに対して日本語版MAC尺度を用いて患者のがんに対する心理的適応（対処様式）を評価する。本尺度はWatosonら^32)^によって開発され、明智ら^33)^により日本語に翻訳され、信頼性と妥当性が確認されている。

　唾液中の成分(salivary cortisol, chromogranin A, s-immunoglobulin A (s-IgA)量の測定は私達の按摩療法の効果の研究^5,18,34)^においてたびたび用いられた測定項目である。

　血液データもマッサージの先行研究ではたびたび用いられてきた。卵巣がん患者に対するマッサージの先行研究では、^35)^CD4, CD8, NK細胞数が増加している。また、Hernandez-Reifらは乳がん患者にマッサージ後、NK細胞やリンパ球数が増加している。^19)^

　酸化ストレスバイオマーカーである尿中8-hydroxydeoxyguanosine(8-OH-dG)を測定することにより、酸化ストレスレベルを推定できる^36)^。生体内では、生命活動を維持するために酸化力の強い活性酸素が常時発生しており、癌を予防するためには体内の酸化ストレスレベルを下げることが必要である^36)^。この指標を用いることで、健康の維持増進に効果があると言われてきた按摩療法ががんサバイバーにとって意義があることが示せる可能性が考えられる。

8.2. 評価スケジュール

　測定は研究開始時と2ヶ月後（研究終了時）に行う。各時点ですべての評価項目を測定し、按摩介入と非介入の比較を行う。自覚症状（VAS）と唾液測定は患者に痛み刺激を与えない簡単な測定であるため、初回治療前後でも行い、按摩施術の直後効果を確認する。その対照として、コントロール群では、研究期間開始時にベッド上での安静40分間をとらせてその前後でVASと唾液測定を行う。

9. データ収集と管理

　本試験に関わる情報・収集したすべてのデータは、連結可能匿名化されたデータベースとして管理する。具体的には、研究の実施により得た対象データは匿名化後、事務局内のパソコンに蓄積されるが、当該パソコンデータへのアクセス権はパスワード認証システムにより限定された関係者のみに与えられる。さらに、個人を特定するための対応表は当該パソコンとは物理的に分離管理された独立した個人情報管理者(研究事務局内共同研究者)のパソコンに格納され、個人情報管理者及びその候補者しか閲覧できない。また、この際に連結する個人情報は、当該パソコンデータとは切り離された如何なるネットワーク上にも存在しないパソコン内データとする。

10. 有害事象の報告

本試験に適用される規制により義務づけられている有害事象の報告等は、研究場所である筑波技術大学の規定に従って施設の責任において適切に行う。

11. 解析対象集団の定義

11.1. 解析対象集団の定義

最終解析で用いる解析対象集団について以下のように定義する。

11.1.1. 全登録例

登録された患者のうち、重複登録や誤登録を除いた集団を「全登録例」とする。学会・論文公表の際の英語表記は、”All randomized”とする。

11.1.2. 全適格例

全登録例から、検討によって決定された「不適格例」を除く集団を「全適格例」とする。

11.1.3. 全治療例

全登録例のうち、プロトコール治療の一部または全部が施行された全患者を「全治療例」とする。

12. 統計学的事項

12.1. 主たる解析と判断規準

本研究では、8週間の按摩継続によるVASの改善効果を評価するために、按摩継続群およびコントロール群における8週間のVAS変化量に対して2標本t検定を実施し、これを主たる解析とする。

Donoyama et. al.(2011) ^18)^の研究では、がんサバイバー5例の施術前におけるVASスコアは、初回施術時の平均値では40.4、8週時の平均値では19.6であった。また、8週時の値から初回施術時の値を引いた変化量の平均値は-20.8であった。もし、按摩１回群（コントロール）において8週間の施術前VASに変化がないと仮定すれば、本試験の結果から、按摩継続により20.8のVAS改善が予想される。しかしながら、上記の推定値は5例のデータに基づくものであること、およびコントロール群においても、心理的要因などの何らかの要因により、VASの変化量に一定の改善傾向が認められる可能性もあることから、実際のVAS改善はこれより小さい可能性も十分想定できる。そこで、本研究では、按摩継続によりVASが10%以上改善した場合に按摩療法は有効である、という臨床的仮説を設定する。但し、後述の通り、VASが約10%改善することを検出力80%以上で検出するためには、試験全体で74例以上の症例数が必要であり、本研究の実施可能性を鑑みると困難であることから、症例数計算ではDonoyama et. al.(2011) ^18)^によって得られたVAS変化量の平均値である-20.8を対立仮説として設定し、かつ後述の感度分析を実施することによって、本研究の評価において十分な検出力を確保した。

VASに対する主解析では、按摩継続群およびコントロール群のVAS変化量（シェーマに記載した測定時点において、按摩継続群：測定E - 測定C、コントロール群、測定H - 測定Fとして定義）に対して2標本t検定を実施し、有意水準両側5％で統計的有意差が得られたときに、施術間に統計的に有意な結果が認められたと宣言する。

なお、本解析の主たる解析対象集団は全治療例であるが、必要に応じて、全登録例および全適格例による解析も感度分析的に実施する。

また、Primary endpointであるVASに対しては、下記の追加解析を実施する：①割付、初回施術時VASを共変量とした共分散分析、②割付、初回施術時VAS、年齢を共変量とした共分散分析③予後因子の可能性が示唆される変数が解析の際に特定されたときは、それをモデルに加えた共分散分析。

さらに、各群における8週間および施術（安静）前後のVAS変化量に対して、対応のあるt検定を実施し、群内における施術前後のVASの変化の有無を統計的に評価するとともに、施術（安静）前後のVAS変化量の差に対して2標本t検定を実施する。

以上の解析で計算されたVAS変化量およびVAS変化量の差に対しては、95％信頼区間を別途計算し、臨床的仮説との比較を実施する。

12.2. 症例数設定の根拠

研究の目標症例数を下記の通り設定する。

がんサバイバー、按摩療法8週実施群：30例

がんサバイバー、按摩療法8週未実施群：30例

計60例

Donoyama et. al.(2011) ^18)^の研究によって得られたデータを、本試験の必要症例数を計算する目的で再解析した。がんサバイバー5例の施術前におけるVASスコアは、初回施術時の平均値は40.4であり、8週時の平均値は19.6であった。また、8週時の値から初回施術時の値を引いた変化量を計算したところ、変化量の平均値は-20.8であり、標準偏差は16.2であった。なお、がんサバイバーにおける施術未実施時のデータは得られていないため、VASの変化量の平均値が0であり、標準偏差が施術実施群と同じ16.2であると仮定した。

前述の通り、主解析として、両群のVAS変化量の差に対して2標本t検定を実施し、有意水準両側5％で統計的有意差が得られたときに、施術間に統計的に有意な結果が認められたと宣言する。このような状況で検出力90%を得るために必要な症例数は1群あたり14例、2群で28例であった。なお、感度分析として、コントロール群におけるVAS変化量の平均値が-5、-7.5および-10であると仮定したときの1群あたりの必要症例数はそれぞれ24例、33例、49例であった。また、検出力80%を確保するための1群あたりの必要症例数はそれぞれ18例、25例、37例であった。以上の計算結果から、主要評価項目に対する主解析の検出力として90％を確保し、また感度分析の多くのシナリオにおいて検出力80％を確保するために、脱落も考慮して、本試験の症例数を1群あたり30例、両群60例と設定した。

研究協力者である筑波大学附属病院産婦人科佐藤豊実が外来管理しているがんサバイバーは200名程度である。予備研究を行った際には協力を呼びかけた6名中5名が登録されたことから、がんサバイバーについては60名の登録は十分な見込みがある。なお、研究期間は筑波技術大学医の倫理委員会承諾後2年間とする。

12.3. Secondary endpointsの解析

　試験の主たる解析結果を補足する考察を行う目的でsecondary endpoints の解析を行う。Secondary endpoints の解析は探索的であるため、多重性の調整は行わない。解析では按摩継続時および単回の按摩（安静）時における各エンドポイントの値に対して、群間比較および群内比較を評価する。具体的には、連続変数に対しては2標本t検定および対応のあるt検定を実施し、カテゴリカル変数に対してはカイ2乗検定を実施するとともに、95%信頼区間を別途計算する。なお、すべての解析における有意水準は両側5%とする。

本解析の主たる解析対象集団は全治療例であるが、必要に応じて全登録例および全適格例による解析も実施する。

12.4. その他の事項

欠測が生じた場合には、原則として欠測例を除いた解析を実施する。なお、感度分析的に欠測値を補完する解析を実施するか否かについては欠測の割合等から総合的に判断する。

統計解析プログラムはSAS9.3（SAS Institute社）を使用する予定である。実際に解析に用いた統計ソフトウェアは論文中に記載する。

13. 倫理的事項

13.1. 患者の保護

　本試験に関係するすべての研究者はヘルシンキ宣言および「臨床研究に関する倫理指針」（平成20年厚生労働省告示第415 号http://www.mhlw.go.jp/general/seido/kousei/i-kenkyu/index.html ）に従って本試験を実施する。本プロトコールでの「医療機関」は、上記指針における「臨床研究機関」に対応する。

13.2. インフォームドコンセント

13.2.1. 患者への説明

　患者登録に先立って、施術担当者は医療機関の承認が得られた説明文書を患者本人に渡し、試験の内容を口頭で詳しく説明する。

13.2.2. 同意

　試験についての説明を行い、十分に考える時間を与え、患者が試験の内容をよく理解したことを確認した上で、試験への参加について依頼する。患者本人が試験参加に同意した場合、付表の同意書を用い、患者本人による署名を得る。同意文書は1部コピーし、患者本人に手渡す。原本は研究事務局が保管する。

13.3. プロトコールの内容変更について

　プロトコール内容変更の際には、変更内容の発効（activation）に先だって「プロトコール改訂申請」を提出し承認を得なければならない。ただし6 か月以内の登録期間の延長は、プロトコール改訂手続き不要とする。

13.4. 補償および患者の経済的負担について

13.4.1. 補償について

　本臨床試験に参加することで生じた健康被害については、通常の診療と同様に病状に応じた適切な治療を保険診療として提供する。その際、医療費の自己負担分については患者の負担とする。また、見舞金や各種手当てなどの経済的な補償は行わない。

13.4.2. 患者の経済的負担について

　通常の経過観察にかかる費用は患者負担とする。

　本試験で必要な血液・唾液の評価項目の測定にかかる費用は研究者負担とする。

研究開始時と終了時（p1シェーマ参照：按摩継続群では、測定A・測定C、按摩1回群では、測定D・測定Fの各2回）に研究事務局内医療機関を受診しなければならず、この際に健康保険による費用を患者が自己負担しなければならない。この経費負担に対して、研究参加終了後に2,000円を補償する。

13.4.3. 研究代表者

　本研究は文部科学省科学研究費補助金平成22～26年度基盤Ｃ『按摩マッサージ療法のエビデンスに基づいた職域の開拓：緩和ケアチームへの参入』（課題番号22531058 研究代表者：殿山　希）の一部分として行うものである。

研究代表者：殿山　希　　筑波技術大学保健科学部保健学科鍼灸学専攻

〒305-8521　茨城県つくば市春日4-12-7

TEL & FAX： 029-858-9631

E-mail： donoyama@k.tsukuba-tech.ac.jp

研究協力者：　佐藤　豊実 筑波大学臨床医学系　産婦人科

〒305-8575　茨城県つくば市天王台1-1-1

TEL： 029-853-3073, FAX： 029-853-3072

E-mail： toyomi-s@md.tsukuba.ac..jp

研究協力者：　大越教夫　　筑波技術大学保健科学部保健学科神経内科

〒305-8521　茨城県つくば市春日4-12-7

TEL & FAX： 029-858-9538

E-mail： ohkoshin@k.tsukuba-tech.ac.jp

研究協力者：　濱野鉄太郎　　株式会社H-STAT　代表取締役

〒158-0082　東京都世田谷区等々力5-11-14

TEL： 03-6326-1958

FAX： 03-6759－3504

E-mail： hamano@h-stat.co.jp

文献

1) 野口栄太郎. 日本にマッサージ技術を導入した人々. 森英俊編: 図解基本術式あん摩・マッサージ・指圧による全身調整 p149-154. 医歯薬出版, 東京 2006.

2) Touch Research Institute http://www6.miami.edu/touch-research/

3) 教科書執筆小委員会.あん摩マッサージ指圧理論.医道の日本社,東京.1988年.

4) 水口公信, 下仲順子, 中里克治訳 (1991) 日本語版STAI使用手引. 京都: 三京房.

5) Donoyama N, Munakata T, Shibasaki M. Effects of Anma Therapy (Traditional Japanese Massage) on Body and Mind. J Bodyw Mov Ther 2010;14:55-64.

6) Donoyama N, Ohkoshi N. Effects of traditional Japanese massage therapy on gene expression (preliminary study). J Altern Complement Med 2011;17(6):1-3.

7) Ministry of Health, Labour and Welfare. Vital statistics. Ministry of Health, Labour and Welfare. www.mhlw.go.jp/toukei/saikin/hw/jinkou/geppo/nengai04/kekka3.html. Accessed Oct 13, 2010.

8) 大野ゆう子,中村隆, 他. 日本のがん罹患の将来推計－ベイズ型ポワソン・コホートモデルによる解析に基く2020年までの予測: がん・統計白書－罹患/死亡/予後/2004. 大島明 ほか編. 篠原出版社, 東京;2004:120-217.

9) 山口建. がん生存者の社会適応に関する研究. 平成13年度厚生労働省がん研究助成金による研究報告集. 国立がんセンター, 東京;2001:276-278.

10) Baile WF, Palmer JL, Bruera E, Parker PA: Assessment of palliative care cancer patients' most important concerns. Support Care Cancer 2010 Apr 17. DOI 10.1007/s00520-010-0839-4, Accessed Apr 22, 2010.

11) Kamibeppu K, Sato I, Honda M, et al.: Mental health among young adult survivors of childhood cancer and their siblings including posttraumatic growth. J Cancer Surviv 2010 Apr 16.DOI 10.1007/s11764-010-0124-z, Accessed Apr 22, 2010.

12) Seitz DC, Besier T, Debatin KM, et al.: Posttraumatic stress, depression and anxiety among adult long-term survivors of cancer in adolescence. Eur J Cancer 2010 Apr 8. DOI 10.1016/j.ejca.2010.03.001, Accessed Apr 22, 2010.

13) Kutner JS, Smith MC, Corbin L, et al.: Massage therapy versus simple touch to improve pain and mood in patients with advanced cancer: a randomized trial. Ann Intern Med 2008;149(6):369-379.

14) Ernst E: Massage therapy for cancer palliation and supportive care: a systematic review of randomised clinical trials. Support Care Cancer 2009;17(4): 333-337.

15) Pruthi S, Degnim AC, Bauer BA, et al.: Value of massage therapy for patients in a breast clinic. Clin J Oncol Nurs 2009;13(4):422-425.

16) Billhult A, Dahlberg K: A meaningful relief from suffering experiences of massage in cancer care. Cancer Nurs.2001;24:180-184.

17) Brauer JA, El Sehamy A, Metz JM, Mao JJ: Complementary and alternative medicine and supportive care at leading cancer centers: a systematic analysis of websites. J Altern Complement Med 2010;16(2):183-186.

18) Donoyama N, Ohkoshi N, Satoh T. Preliminary Study on the Physical and Psychological Effects of Traditional Japanese Massage Therapy in Cancer Survivors. J Jpn Assoc Phys Med Baln Clim 2011;74(3):155-167.

19) Hernandez-Reif M, Ironson G, Field T: Breast cancer patients have improved immune and neuroendocrine functions following massage therapy. J Psychosom Res 2004;57(1):45-52.

20) Brown LF, Kroenke K, Theobald DE, et al.: The association of depression and anxiety with health-related quality of life in cancer patients with depression and/or pain. Psychooncology 2010 Jul 19.DOI: 10.1002/pon.1627, Accessed Apr 15, 2010.

21) Deng G, Cassileth BR: Integrative oncology: complementary therapies for pain, anxiety, and mood disturbance. CA Cancer J Clin 2005;55(2):109-116.

22) Campeau MP, Gaboriault R, Drapeau M, et al.: Impact of massage therapy on anxiety levels in patients undergoing radiation therapy: randomized controlled trial. J Soc Integr Oncol 2007;5(4):133-138.

23) Zigmond AS, Snaith RP. The hospital anxiety and depression scale. Acta Psychiatrica Scandinavica. 1983; 67(6): 361-70.

24) Zigmond AS, Snaith RP, 北村俊則. Hospital anxiety and depression scale(HAD尺度). 精神科診断学. 1993; 4(3): 371-2.

25) Kugaya A. Akechi T. Okuyama T. Okamura H. Uchitomi Y. Screening for psychological distress in Japanese cancer patients. Japanese Journal of Clinical Oncology. 1998; 28(5): 333-8.

26) Keir ST. Effect of massage therapy on stress levels and quality of life in brain tumor patients--observations from a pilot study. Support Care Cancer. 2011;19(5):711-5.

27) Aaronson NK, Ahmedzai S, Bergman B, Bullinger M, Cull A, Duez NJ, et al. The European Organization for Research and Treatment of Cancer QLQ-C30: a quality-of-life instrument for use in international clinical trials in oncology. Journal of the National Cancer Institute. 1993; 85(5): 365-76.

28) 下妻晃二郎、江口成美：がん患者用QOL尺度の開発と臨床応用（I）．日医総研ワーキングペーパー　No. 56　2001年11月

29) Lazarus R, Folkman S. Stress, Appraisal, and Coping. New York: Springer Publishing Co; 1984.

30) Watson M, Greer S, Rowden L et al. 前掲論文37). 1991.

31) Akechi T, Okuyama T, Imoto S et al. Biomedical and Psychosocial Determinants of Psychiatric Morbidity Among Postoperative Ambulatory Breast Cancer Patients. Breast Cancer Research and Treatment. 2001; 65 (3): 195-202.

32) Watoson M, Greer S, Young J, et al. Development of a Questionaire measure of adjustment to

cancer: the MAC scale. Psychol Med. 1988; 18: 203-9.

33) 明智龍男, 久賀谷亮, 岡村仁, et al. Mental Adjustment to Cancer (MAC) scale 日本語版の信頼性・妥当性の検討.精神科治療学 . 1997; 12: 1065-71.

34) Donoyama N, Shibasaki M. Differences in practitioners' proficiency affect the effectiveness of massage therapy on physical and psychological states. J Bodyw Mov Ther 2010;14:239-244.

35) Judson PL, Dickson EL, Argenta PA, Xiong Y, Geller MA, Carson LF, Ghebre R, Jonson AL, Downs LS Jr. A prospective, randomized trial of integrative medicine for women with ovarian cancer. Gynecol Oncol. 2011;123(2):346-50.

36) 越智宏倫, 蔵重淳.生体内酸化ストレス評価と癌予防：酸化ストレスプロファイル.医学のあゆみ2003;204(1):25-31.

資料１

先行論文より引用

Donoyama N, Munakata T, Shibasaki M. Effects of Anma Therapy (Traditional Japanese Massage) on Body and Mind. J Bodyw Mov Ther 2010;14:55-64.

Outline of the procedure of the Anma therapy is described briefly in the following:

Anma therapy is performed usually through clothing or using a piece of cloth.

I Lying down on one side for 17 minutes

1. Procedure for the back including the shoulder, the back, and the lower back

(1) Stroking starts at the base of the neck along the upper shoulder to the shoulder joint

(2) Downward strokes along the full length of the back, starting at the base of the neck down to the waist

(3) Thumb kneading by circular or linear (back and forth) movement: the upper shoulder from the side of the 7th cervical vertebra (Cv7) to the *acromion* along the *trapezius*

(4) Thumb kneading by circular or linear movement: from Cv7 via the superior angle of the *scapula* and the *supraspinous fossa* to the *acromion*, on the *levator scapulae*, *rhomboid*, and *supraspinatus*.

(5) Thumb kneading by linear movement along the spine: from the side of Cv7 to the side of the 5th lumbar vertebrae (Lv5) on the *erector spina*e and the *quadratus lumborum*

(6) Thumb or other four-finger kneading by circular or linear movement on the *medial* and *lateral border of scapula*

(7) Heel of the hand kneading by circular movement on the *infraspinous fossa*

(8) Downward strokes again along the length of the back, starting at the base of the neck down to the waist

(9) Stroking again starting at the base of the neck along the upper shoulder to the shoulder joint

2. Procedure for the upper limb and the hand

(1) Stroking down from the shoulder to the fingertips

(2) Palm grasp kneading over the upper limb on the *deltoid*

(3) Palm grasp kneading over the upper front limb on the *biceps brachii*

(4) Palm grasp kneading over the back of the upper limb on the *triceps brachii*

(5) Thumb kneading on the back of the forearm

(6) Palm grasp kneading on the front and the side of the forearm

(7) Thumb kneading on the hand

(8) Knead and squeeze each finger along the full length using the thumb and the index (9) Stroking down from the shoulder to the fingertips

3. Procedure for the neck

(1) Stroking starting at the superior nuchal line along the neck to the base of the neck

(2) Thumb kneading over the back of the neck on the *semispinal capitis*, the *splenius capitis*, and the *trapezius* descending part

(3) Thumb, two-finger (thumb and index), or four-finger kneading to the side of the neck, on the *sternocleidomastoid*

(4) Apply four-finger kneading to the front of the neck

(5) Thumb kneading and pressure along the superior nuchal line

(6) Stroking again starting at the superior nuchal line along the neck to the base of the neck

4. Start again the cycle for item 1.

5. Procedure for the lower limb and the foot

(1) Stroking from the buttock to the toes

(2) Kneading over the buttock with the heel of the hand

(3) Palm kneading on the front thigh, on the *quadriceps femoris* muscle

(4) Palm grasp kneading to the back thigh or hamstrings

(5) Palm grasp kneading on the patella

(6) Thumb kneading on the front lower leg

(7) Palm grasp kneading on the calf muscles

(8) Palm grasp kneading of the Achilles tendon

(9) Finger kneading over the top of the foot

(10) Thumb kneading and pressure on the sole

(11) Knead and squeeze each toe along the length using thumb and index finger.

(12) Intermittent palm pressure on the entire leg

(13) Stroking again from the buttock to the toes

II Lying down on the opposite side, repeat 1 to 5 for 17 minutes

III Conclusion: in the prone position for 6 minutes

(All the techniques in this conclusion section are done simultaneously on the left and right side of the subject.)

(1) Stroking starts at the superior nuchal line along the sides of the neck and the upper shoulders to the shoulder joints

(2) Downward strokes along the full length of the back, starting at the base of the neck down to the waist

(3) Grasp hand kneading, thumb kneading, and pressure over the back of the neck

(4) Four-finger kneading and pressure on the sides of the neck

(5) Grasp hand kneading, thumb kneading, and pressure on the upper shoulder

(6) Thumb kneading and pressure along the spine

(7) Palm grasp hand kneading over the sides of the back, from the waist to the shoulder joints  *latissimus dorsi*

(8) Downward strokes again along the full length of the back

(9) Stroking again from the superior nuchal line along the sides of the neck and the upper shoulders to the shoulder joints
